# Molecular characterisation of human Shiga toxin-producing *Escherichia coli* O26 strains: results of an outbreak investigation, Romania, February to August 2016

**DOI:** 10.2807/1560-7917.ES.2017.22.47.17-00148

**Published:** 2017-11-23

**Authors:** Codruţa-Romaniţa Usein, Adriana Simona Ciontea, Cornelia Mãdãlina Militaru, Maria Condei, Sorin Dinu, Mihaela Oprea, Daniela Cristea, Valeria Michelacci, Gaia Scavia, Lavinia Cipriana Zota, Alina Zaharia, Stefano Morabito

**Affiliations:** 1 Cantacuzino National Institute of Research, Bucharest, Romania; 2 Carol Davila University of Medicine and Pharmacy, Bucharest, Romania; 3 Food Safety, Nutrition and Veterinary Public Health Department, Istituto Superiore di Sanità, Rome, Italy; 4 National Center for Surveillance and Control of Communicable Diseases, National Institute of Public Health, Bucharest, Romania

**Keywords:** Shiga toxin-producing E. coli, STEC, STEC O26, laboratory surveillance, molecular methods, food-borne infections, haemolytic uremic syndrome

## Abstract

At the beginning of 2016, an increase in paediatric haemolytic uremic syndrome (HUS) cases was observed in Romania. The microbiological investigations allowed isolation of Shiga toxin-producing *Escherichia coli* (STEC) O26 as the causative agent from most cases. **Methods:** An enhanced national surveillance of HUS and severe diarrhoea was established across the country following the identification of the first cases and was carried out until August 2016. A total of 15 strains were isolated from 10 HUS and five diarrhoea cases. Strains were characterised by virulence markers (i.e. *stx* type/subtype, *eae*, *ehxA* genes), phylogroup, genetic relatedness and clonality using PCR-based assays, PFGE and multilocus sequence typing (MLST). The first six strains were further characterised by whole genome sequencing (WGS). **Results:** Five PCR-defined genotypes were distinguished. All strains from HUS cases harboured *stx2a* and *eae*, with or without *stx1a*, while strains from diarrhoea cases carried exclusively *stx1a* and *eae* genes. PFGE resolved strains into multiple pulsotypes, compatible with a certain geographic segregation of the cases, and strains were assigned to phylogroup B1 and sequence type (ST) 21. WGS confirmed the results of conventional molecular methods, brought evidence of O26:H11 serotype, and complemented the virulence profiles. **Discussion/conclusion:** This first description of STEC O26 strains from cases in Romania showed that the isolates belonged to a diverse population. The virulence content of most strains highlighted a high risk for severe outcome in infected patients. Improving the national surveillance strategy for STEC infections in Romania needs to be further considered.

## Introduction

The role of Shiga toxin-producing *Escherichia coli* (STEC) in outbreaks of food-borne illnesses is well recognised [1]. According to surveillance studies, STEC of serotype O157:H7 are those most often associated with epidemics of STEC infections worldwide [2-4] and most likely to cause severe infections with systemic complications such as haemolytic uremic syndrome (HUS) [5]. Nonetheless, STEC belonging to non-O157 serogroups raised public health concern in many countries by displaying the same pathogenic potential as O157:H7 [6-8]. Among them, STEC O26 poses a significant threat to human health in terms of illness severity and the risk of causing outbreaks. This serogroup is considered emerging in Europe and in the last decade, it was reported as the most frequent non-O157 STEC serogroup in human sporadic cases of infection, including those developing HUS [9]. Severe outbreaks of STEC O26 infection have been reported both in community [6] and childcare settings [10]. In 2013, the emergence of a highly virulent clone of STEC O26 strongly associated with HUS in Europe was described in the scientific literature [11].

At the beginning of 2016, Romania alerted the European Centre for Disease Prevention and Control (ECDC) of an unprecedented rise in haemolytic uremic syndrome (HUS) cases in the paediatric population. It reported on 15 children aged between 5 and 38 months who were diagnosed with HUS, some of whom progressed to HUS after bloody or non-bloody diarrhoea. Cases were linked to STEC O26 infection by serology and most resided in one of the southern districts [12]. Food consumption history of cases suggested that dairy products from a local producer were among the foodstuff potentially implicated in the outbreak as the source or vehicle of infection, but this suspicion was not confirmed microbiologically. Soon after the description of the first cases in Romania, Italy reported one HUS case with epidemiological link to Romania through the Early Warning and Response System (EWRS) of the European Union, suggesting that a multi-country outbreak of STEC infections was ongoing [13].

Microbiological characterisation and typing were carried out on STEC strains isolated from children with HUS, bloody or non-bloody diarrhoea identified through the ad hoc enhanced surveillance established in Romania following the identification of the outbreak. The aim was to provide insight into the epidemiology of STEC O26 infection. The goal of the presented microbiological investigation was to provide evidence for the national public health authorities of the relevance of adopting typing/subtyping strategies to support surveillance of STEC infection in humans.

## Methods

### Strain collection

**Figure 1:**
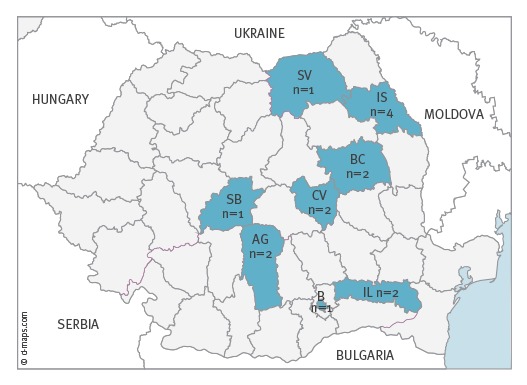
Geographic distribution of the children with culture-confirmed Shiga toxin-producing *Escherichia coli* O26 HUS, bloody or non-bloody diarrhoea included in the study, Romania, 2016 (n = 5)

AG: Arges; B: Bucharest; BC: Bacau; CV: Covasna; HUS: haemolytic uremic syndrome; IL: Ialomita; IS: Iasi; SB: Sibiu; SV: Suceava.

Fifteen STEC O26 strains that were recovered from the stool samples of 15 children hospitalised in Romania for HUS (10 cases), bloody diarrhoea (one case) or non-bloody diarrhoea (four cases) were characterised in this study. The children had a median age of 12 months (range 6–24 months) and the male to female ratio was 1.14. They resided in several districts across the country (Figure 1). The strains were given identification numbers from 1 to 15 in accordance with the chronological order of isolation (Table 1).

**Table 1:** Clinical and epidemiological information associated with the Shiga toxin-producing *Escherichia coli* O26 strains isolated from the stool samples of children, Romania, 2016 (n = 15)

Strain identification number^a^	Month of isolation	Clinical diagnostic	District of residence
1^b^	February	HUS	Bacau
2^b^	February	HUS	Arges
3^b^	February	BD	Sibiu
4^b^	February	HUS	Bucharest
5^b^	March	HUS	Ialomita
6^b^	March	HUS	Ialomita
7	April	D	Suceava
8	April	HUS	Iasi
9	April	D	Iasi
10	April	HUS	Iasi
11	May	HUS	Arges
12	May	HUS	Bacau
13	May	HUS	Iasi
14	May	D	Covasna
15	June	D	Covasna

BD: bloody diarrhoea; D: diarrhoea; HUS: haemolytic uremic syndrome.

^a^ The strains were given identification numbers from 1 to 15 in accordance with the chronological order of isolation.

^b^ Strain subjected to whole genome sequencing.

### Shiga toxin-producing *Escherichia coli* O26 characterisation

The strains, biochemically confirmed as *E. coli*, were typed as O26 using slide agglutination with commercially available OK O pool and single antisera (SSI Diagnostica, Hillerød, Denmark). Phenotypical test for H antigen identification was not performed. The presence of genes considered as hallmarks for the STEC pathotype was assessed using a multiplex PCR-based commercial kit (DEC Primer Mix, SSI Diagnostica, Hillerød, Denmark). The kit contained a mix of primers directed towards *stx1* (Shiga toxin 1), *stx2* (Shiga toxin 2), *eae* (intimin), *eltA* (heat-labile enterotoxin), *estA* (heat-stable enterotoxin, human and porcine variants), and *ipaH* (invasive plasmid antigen) genes, suited for the distinction between STEC, enteropathogenic *E. coli* (EPEC), enterotoxigenic *E. coli* (ETEC) and enteroinvasive *E. coli* (EIEC) strains. Additional commercially available primers (EAEC PCR kit, SSI Diagnostica, Hillerød, Denmark) were used to detect enteroaggregative *E. coli* (EAEC)-associated genes *attA, aggR, aap,* and *aaiC*. Enterohaemolysin-encoding gene *ehxA* was identified using primers described by Schmidt et al. [14].

### *stx* gene subtype detection by conventional PCR

The *stx1* and *stx2* genes were subtyped using published primers and protocols [15]. The complete set of strains harbouring the genes encoding all *stx* subtypes, which were used as PCR positive controls, were kindly provided by the World Health Organization (WHO) Collaborating Centre for Reference and Research on *Escherichia* and *Klebsiella* from Statens Serum Institute, Denmark.

### Phylogenetic group typing, PFGE, and multilocus sequence typing

Phylogenetic group assignment of the STEC O26 strains was performed according to the method described by Clermont et al. [16].

PFGE was performed using the protocol approved by ECDC [17] and the cluster analysis was performed with BioNumerics software (Version 6.6, Applied Maths, Sint-Martens-Latem, Belgium). DNA fragments smaller than 33 kb were not included in the analysis. The dendrogram of similarity was generated using the band-based Dice similarity coefficient (with 1.5% band position and 1.5% optimisation tolerance), and the unweighted pair group method with arithmetic mean (UPGMA). Clusters were defined as a group of profiles sharing ≥ 93% similarity, corresponding to a ca 3-bands difference [18].

All study strains were subjected to standard multilocus sequence typing (MLST) with primers and protocols specified at the *E. coli* MLST website [19].

### Whole genome sequencing and bioinformatic analysis

An Ion Torrent Personal Genome Machine (Thermo Fisher Scientific, Waltham, United States (US)) was used for the whole genome sequencing (WGS) of six STEC O26 strains obtained from the first culture-confirmed cases of infection. Five of the sequenced strains (i.e. strains 1, 2, 4–6) were isolated from stool specimens of children with HUS and one (i.e. strain 3) was isolated from the faecal sample of a child hospitalised with gastrointestinal symptoms.

The DNA was extracted using PureLink Genomic DNA Mini Kit (Invitrogen, Thermo Fisher Scientific, Waltham, US), according to the manufacturer’s protocol. Enzymatic shearing of genomic DNA samples and generation of barcoded libraries were carried out using the Ion Xpress Plus Fragment Library Kit and Ion Xpress Barcode Adapters Kit (Thermo Fisher Scientific, Waltham, US). Libraries were size-selected by electrophoresis using E-Gel SizeSelect 2% gels (Thermo Fisher Scientific, Waltham, US) and templates were prepared using the Ion PGM Hi-Q OT2 Kit and the Ion OneTouch 2 System. Sequencing was conducted on an Ion 318 chip Kit using the Ion PGM Hi-Q 400bp Sequencing Kit (Thermo Fisher Scientific, Waltham, US). The sequence raw data were analysed using the tools present in the Advanced Research Infrastructure for Experimentation in genomics (ARIES) public Galaxy server [20].

The raw reads were trimmed to remove the adaptors and to accept 25 as the lowest Phred value. The detection of the virulence genes was performed through the virulotyper pipeline, which performs mapping of sequencing reads on the database of sequences of *E. coli* virulence genes [21] using the Bowtie2 tool [22]. Typing of the intimin-coding genes was performed in silico by comparing the sequence of the *eae* allele identified in the database against the National Center for Biotechnology Information (NCBI) Reference Sequence Database (RefSeq).

The trimmed reads were subjected to de novo assembly using the tool SPADES with default parameters (k-mers length set at 21, 33 and 55) [23]. The obtained contigs were filtered to accept a minimum length of 1,000 bp and used 10 as coverage cut-off. The NCBI Basic Local Alignment Search Tool (BLAST) + blastn tool was used to determine the serotype through alignment of the assembled contigs with the database containing the sequences of serotype-associated genes of pathogenic *E. coli* [24]. In silico MLST was performed by interrogating the database downloaded from the MLST website [19] using the SRST2 software [25].

**Figure 2:**
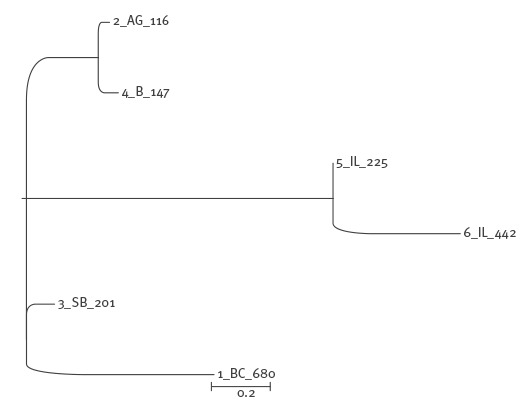
Phylogenetic analysis of six Shiga toxin-producing *Escherichia coli* O26 strains part of the outbreak according to whole genome single nucleotide polymorphisms (WG SNP) analysis, Romania, 2016

Whole genome single nucleotide polymorphisms (WG SNP) analysis was performed using the kSNP3 pipeline [26] and 19 as kmer size. The optimum value for the kmer size was selected as that producing the highest number of unique kmers of the median length in all the genomes of the dataset, and was calculated by using the kchooser tool included in the kSNP3 pipeline [26]. The dendrogram was obtained by using the maximum likelihood clustering algorithm [27] (Figure 2).

The tree was obtained by using the kSNP3 tool to identify the total SNPs in the set of sequences analysed. The phylogenetic tree was estimated based upon those SNPs using the Maximum Likelihood clustering algorithm. The names of the strains include the strain identification number, the abbreviation for the district of residence of the child from whom the strain was isolated and the strain specific alleles count, each separated by an underscore. The scale of 0.2 indicates the branch lengths, expressed in terms of changes per number of SNPs.

The sequencing reads and the corresponding assembled contigs have been deposited in the European Molecular Biology Laboratory (EMBL)-European Nucleotide Archive (ENA) sequence database (Accession number PRJEB19376).

## Results

### PCR-based virulotyping of Shiga toxin-producing *Escherichia coli* O26 strains

**Figure 3:**
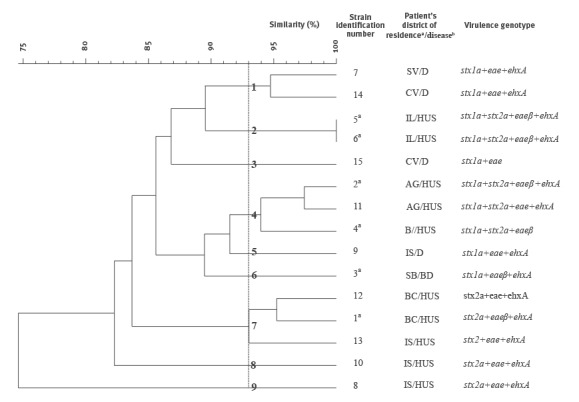
Dendrogram of *Xba*I PFGE profiles showing the genetic relatedness of Shiga toxin-producing *Escherichia coli* O26 strains and associated bacterial virulence genes, Romania, 2016

AG: Arges; B: Bucharest; BC: Bacau; BD, bloody diarrhoea; CV: Covasna; D: diarrhoea; HUS: haemolytic uremic syndrome; IL: Ialomita; IS: Iasi; SB: Sibiu; SV: Suceava.

^a^ Strain subjected to whole genome sequencing.

The study collection included 15 STEC O26 strains isolated from cases with HUS (n = 10), bloody diarrhoea (n = 1) or non-bloody diarrhoea (n = 4) reported across Romania between February and June 2016. PCR assays showed that 10 strains were positive for *stx2* gene, either alone (n = 5) or in combination with *stx1* (n = 5), while the remaining five were positive for *stx1* gene alone. Subtyping of the *stx* genes showed that the *stx1*-positive strains carried *stx1a* subtype and the *stx2*-positive strains harboured *stx2a* subtype. The *eae* gene was found in all the strains and *ehxA* in all but two of them (Figure 3). While *eae* and *ehxA* genes were detected irrespective of the strain origin, a difference in the distribution of the Shiga toxin-coding genes was observed. In fact, *stx2* gene alone or associated with *stx1* was found only in HUS-associated strains while *stx1* as sole *stx* gene was detected only in the strains recovered from children with diarrhoea. No other pathogenic *E. coli* virulence genes were identified.

### Phylogenetic group typing, PFGE and multilocus sequence typing

The STEC O26 strains assayed in this study were classified into phylogenetic group B1 and sequence type ST21. All the strains yielded interpretable *Xba*I PFGE profiles that clustered on the dendrogram with 74.5% overall similarity (Figure 3). The strains were assigned to 14 different pulsotypes all but one including only one isolate. The comparative analysis showed five single pulsotype clusters (strain identification numbers 3, 5, 6, 8, and 9) and four multiple-pulsotype clusters showing over 93% of similarity (strain identification numbers 1, 2, 4, and 7). Two of the latter contained three strains each (Figure 3). Not all the strains belonging to multiple-strain clusters could be assigned to similar geographic areas. Nevertheless, the two strains showing an indistinguishable profile (pulsotype 2) were recovered from cases from the same district. Also clusters 4 and 7 contained strains isolated from cases residing in the same district and in a neighbouring one (i.e. Iasi and Bacau; Arges and Bucharest) (Figure 1).

### Genomic characterisation and typing

In silico WGS-based O and H serotyping assigned the six sequenced strains to the serotype O26:H11, a piece of information that complemented the conventional serotyping which was restricted to O antigen identification. Moreover, WGS confirmed that all these strains were indeed members of ST21 clone.

The results of the virulotyper confirmed the genotypes identified through PCR and additionally identified accessory virulence genes in six strains that were analysed (Table 2). The comparison of the *eae* gene sequence identified in the tested strains with those in the NCBI Reference Sequence Database (RefSeq) allowed identification of the type β intimin allele in all the genomes. A limited variation in the gene content was observed among the sequenced isolates mainly for genes usually located on plasmids. In detail, only two isolates harboured the plasmid-borne *cba* gene, encoding colicin B. One strain lacked the *ehxA, espP, katP* and *toxB* genes, described to be conveyed by the virulence plasmid of STEC O26 (Table 2) and that were present in all the other genomes analysed.

**Table 2:** Virulence genes identified in the Shiga toxin-producing *Escherichia coli* O26 strains subjected to whole genome sequencing, Romania, 2016

Strain identification number	Virulence genes																						
	*cba*	*cif*	*eae*	*efa1*	*ehxA*	*epeA*	*espA*	*espB*	*espF*	*espJ*	*espP*	*gad*	*iha*	*iss*	*katP*	*lpfA*	*nleA*	*nleB*	*nleC*	*stx1a*	*stx2a*	*tir*	*toxB*
1	–	+	+	+	+	–	+	+	+	+	+	+	+	+	+	+	+	+	+	–	+	+	+
2	–	+	+	+	+	–	+	+	+	+	+	+	+	+	+	+	+	+	+	+	+	+	+
3	–	+	+	+	+	–	+	+	+	+	+	+	+	+	+	+	+	+	+	+	–	+	+
4	–	+	+	+	–	–	+	+	+	+	–	+	+	+	–	+	+	+	+	+	+	+	–
5	+	+	+	+	+	–	+	+	+	+	+	+	+	+	+	+	+	+	+	+	+	+	+
6	+	+	+	+	+	+	+	+	+	+	+	+	+	+	+	+	+	+	+	+	+	+	+

+: present; –: absent.

The whole genome SNPs comparison allowed for producing a dendrogram with topology compatible with that obtained through PFGE typing. The high resolution of the SNPs analysis allowed identification of differences between the strains that appeared indistinguishable using PFGE (Figure 2). In detail, two clear clusters were identified in the dendrogram produced from the results of the SNPs analysis (Figure 2), one including the strains 5 and 6 (cluster 2 in Figure 3) and a second one grouping the strains 2 and 4 (part of cluster 4 in Figure 3).

## Discussion

This study focuses on the microbiological characterisation of STEC O26 strains isolated from cases identified in Romania during a community-wide outbreak of STEC-associated HUS [13]. The combined use of microbiological and serological techniques, the latter performed with ECDC support, brought evidence that STEC belonging to serogroup O26 were the cause of infection in most of the patients involved in the outbreak [13].

An outbreak was suspected because of the high number of HUS cases observed in a short period of time (January and February 2016) among children from Arges district in Romania; this was a twofold increase compared with cases reported in the six previous years by four regional Romanian hospitals, Bucharest, Cluj, Iasi and Timisoara [13]. This triggered a more thorough investigation on STEC infection in the country. Active case finding was promoted at the national level by the public health authorities on 15 February 2016 and resulted in the identification of cases that could have remained undetected. The enhanced surveillance based on the reporting of cases presenting with HUS or severe diarrhoea to the national health authorities revealed that STEC cases had a wide geographical distribution, with cases in seven districts in addition to those in Arges.

Epidemic cases were defined according to the ECDC case definition [13]. Before outbreak onset, difficulties in monitoring HUS across the country were due to the lack of mandatory reporting of HUS as well as the limited familiarity of many physicians with the clinical course of STEC infection and HUS owing to the rare occurrence of this condition.

STEC O26 strains were obtained from patients involved in the outbreak across the country. One of the strains, strain 2, was isolated from a child from Arges district with a history of exposure to the dairy products initially suspected to be implicated in the transmission of STEC O26 infection to the epidemic cases. The rest of the strains originated from children with no obvious exposure to such products.

All the STEC O26 strains were characterised using molecular typing methods allowing identification of virulence determinants and the clonal relationship. Virulence genes typing showed that the strains from all the children with HUS carried the *stx2a* gene, which is common in STEC O26 associated with HUS, especially in small children under 5 years of age [3,28-30]. By contrast, STEC O26 strains harbouring only *stx1,* usually configuring a profile of less severe clinical outcome [3,28,31], were found in children who did not progress to HUS. Irrespective of the clinical features, all the STEC O26 strains possessed the intimin-coding gene *eae* which indicated that these strains were capable of producing attaching and effacing lesions in the intestinal mucosa [32]. Additionally, most strains were positive for enterohaemolysin-coding gene *ehxA*, a marker of the large virulence plasmids associated with the most pathogenic STEC strains involved in human illness [33,34]. The first six strains recovered during the outbreak were further characterised through WGS, which allowed identification of additional virulence genes, type all the sequenced strains as O26:H11, and the intimin coding gene as *eae*β. Unfortunately, due to limited resources and time constraints, the rest of the STEC O26 strains could not be sequenced prior to manuscript submission.

The Romanian strains studied were exclusively typed as ST21, the clone that includes the majority of human STEC O26 strains isolated from clinical cases of STEC infection in Europe [11]. With one exception (i.e. strain 4), the sequenced strains were also shown to harbour the classic plasmid gene profile described in the vast majority of STEC O26 belonging to ST21 [11], including positivity for the three virulence genes *exhA*, *katP* and *espP*. The sequence type and the plasmid profile indicate that the strains analysed did not belong to the emerging STEC O26 ST29 clone, which has been increasingly described in Europe [11,35,36].

When analysing strains through PFGE and WGS, they were found to belong to a heterogeneous population. Interestingly, strains with the highest similarity in PFGE (> 97.4%) were isolated from patients residing in the same district (strains 5, 6 from Ialomita patients and strains 2, 11 from Arges patients) suggesting a link among some cases. The heterogeneity of the isolates suggested that the outbreak had a possible multi-clonal aetiology, similarly to the STEC O26 outbreak observed in Italy in 2013 [6] However, the heterogeneity of isolates could also reflect the low specificity of the confirmed case definition adopted at the European level [13], possibly leading to a misclassification of sporadic cases of STEC O26 infection as part of the outbreak.

Multiple aetiology outbreaks (i.e. outbreaks with multiple causative agents or multiple clones belonging to the same agent) are often reported in community-wide outbreaks of non-O157 STEC infection [8]. This highlights the importance of applying molecular typing techniques that are able to discriminate the clonal relationship of strains belonging to the same STEC serotypes, especially for those that are most frequently reported in humans.

A possible selection bias of the STEC O26 strains subjected to characterisation should be mentioned. The strains in this study were not necessarily representative of all the incident cases of STEC O26 infections occurring in Romania during the epidemic period as cultures from the earliest HUS cases were not available and milder clinical cases could have been overlooked. On the other hand, the opportunity to examine a set of clinical isolates from several regions of Romania and the results from the extensive molecular characterisation, including whole genome sequencing, served as reference data for Romanian STEC O26 strains and are suitable for comparison with similar human, food or animal isolates at a national or international level.

The cause(s) of the outbreak observed in Romania remained unknown in spite of evidence of an epidemiological link with the consumption of contaminated dairy products from local producers [13]. None of the dairy products sampled in Romania tested positive for STEC [13]. Interestingly, during the epidemic period in Romania, one case of HUS caused by STEC O26 infection and epidemiologically linked to the cases in Romania was described in Italy [13].

In conclusion, this study provides data on the characterisation of STEC O26 strains isolated from human cases of disease in Romania for first time and it describes a rather genetically heterogeneous population of STEC O26 belonging to the ST21 clone, and possessing virulence gene combinations able to cause severe condition such as HUS.

The 2016 outbreak clearly showed the need to rapidly detect and characterise the STEC strains causing human diseases with molecular typing techniques in order to understand their epidemiology and circulation, and thereby support targeted measures that limit human exposure to the source of infection. As part of an effective strategy for the control of STEC infection in the population, the adoption of a sensitive national surveillance system for STEC infection and HUS is desirable. The system should be capable of providing data on the incidence of HUS and STEC infection at both the national and regional level, and describing the characteristics of both clinical cases and STEC strains. The timely subtyping and assessment of virulence profiles of STEC strains isolated from patients would facilitate the early detection of outbreaks in the communities. For future research the whole genome sequence data will be used to understand the genetic diversity and clonal relatedness of the STEC O26 strains isolated from patients in Romania and in Italy during the 2016 outbreak.
